# Ileal perforation secondary to bowel obstruction caused by foreign body bezoar: A case report

**DOI:** 10.1016/j.amsu.2022.104564

**Published:** 2022-09-05

**Authors:** Nischal Shrestha, Sujan Regmee, Abhiyan Kharel, Mandeep Guragai

**Affiliations:** a Department of Surgery, Kathmandu Medical College and Teaching Hospital, Sinamangal, Kathmandu, Nepal; b Kathmandu Medical College and Teaching Hospital, Sinamangal, Kathmandu, Nepal

**Keywords:** Bezoars, Case reports, Ileocecal valve, Surgical anastomosis

## Abstract

**Introduction and importance:**

Foreign body bezoar is a relatively uncommon variant of bezoars leading to intestinal obstruction and perforation. These are caused by the ingestion of indigestible materials that gradually grow in size.

**Case presentation:**

Following is the case of a young female patient with abdominal pain and distension which was associated with nausea, vomiting, and obstipation. Contrast-enhanced computed tomography of the abdomen showed dilated jejunal and ileal loops, and a tubular hypodense structure on terminal ileum. During surgery, we discovered intraluminal foreign bodies and ileal perforation proximal to the ileocecal valve. The findings were suggestive of obstruction and perforation of terminal ileum secondary to foreign body obstruction. The patient was managed successfully with ileocolic resection and anastomosis.

**Discussion:**

Patients with bezoars can remain asymptomatic or present with features of bowel obstruction. These are usually discovered while performing radiological imaging for the evaluation of symptoms. Though mild to moderate cases of bezoars resolve with the treatment by chemical dissolution, surgeries should be performed in patients with foreign body bezoars and in whom complications have arose.

**Conclusion:**

Ingested foreign body could lead to formation of a bezoar which may cause obstruction and perforation—the sequelae must be kept in mind while managing a patient.

## Highlights

• Foreign body bezoars are rare causes of bowel obstruction and perforation.
• High index of suspicion must be kept while evaluating patients with psychiatric illnesses.
• Radiology helps in identifying foreign body as the cause.
• Bezoars should be considered as one of the differentials of bowel obstruction when the exact etiology is unclear.

## 1 Introduction

Bezoars are intraluminal conglomerates of indigestible foreign materials that accumulate in the gastrointestinal tract [1]. Although rare in itself, the stomach is a common site for the formation of bezoars, which could migrate to the small intestine and cause obstruction [2]. However, an isolated foreign body bezoar in the small bowel is a rare clinical scenario. Diagnosis is based on thorough history of foreign body ingestion and subsequent history suggestive of bowel obstruction and or perforation [3]. Physical examination findings including abdominal tenderness can provide supportive evidence for diagnosis and radiology could reveal the etiology [3]. Both ultrasound and contrast-enhanced computed tomography (CECT) are reliable methods with CECT being more accurate [2]. However, endoscopy is advantageousin early stages—when the bezoar is in the upper gastrointestinal tract, as it provides the advantage of direct visualization and occasionally has therapeutic applications [4]. We present a case of a young female presenting with bowel perforation and a history of foreign body ingestion successfully managed with surgical intervention. This case report has been prepared in line with the Surgical Case Report (SCARE) guidelines [5].

## 2 Presentation of case

A 22-year-old female presented to the emergency department of our center with a history of foreign body ingestion 8 days back. It was followed by an initial colicky abdominal pain in the periumbilical region, which was later generalized at presentation. The pain was associated with nausea, vomiting, abdominal distention, and fever. The patient reported that she hadn't passed feces or flatus. Past medical or surgical history of the patient was insignificant.

On clinical evaluation, vitals were stable. Per abdominal examination revealed a distended abdomen with decreased movement with respiration. On palpation, the abdomen was tense, tender, and with guarding and rigidity but no palpable mass was felt. Furthermore, hypoactive bowel sounds were heard on auscultation. Based on the clinical evaluation, we made diagnosis of intestinal obstruction with a possibility of bowel perforation. The patient was sent for radiological investigation.

Contrast Enhanced Computed Tomography (CECT) scan of the abdomen showed dilated jejunal and ileal loops. A tubular hypodense structure with surrounding mixed density areas was seen in the terminal ileum. It extended up to the ileal walls with mild thickening of the walls. Minimal collection adjacent to this area was seen. The findings were suggestive of probable inflammation and obstruction of the small bowel due to a foreign body.

**Fig. 1:**
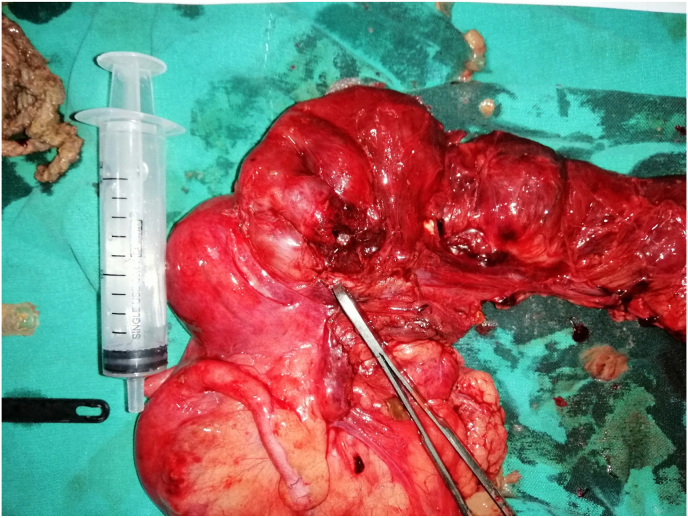
Resected ileocolic specimen.

**Fig. 2:**
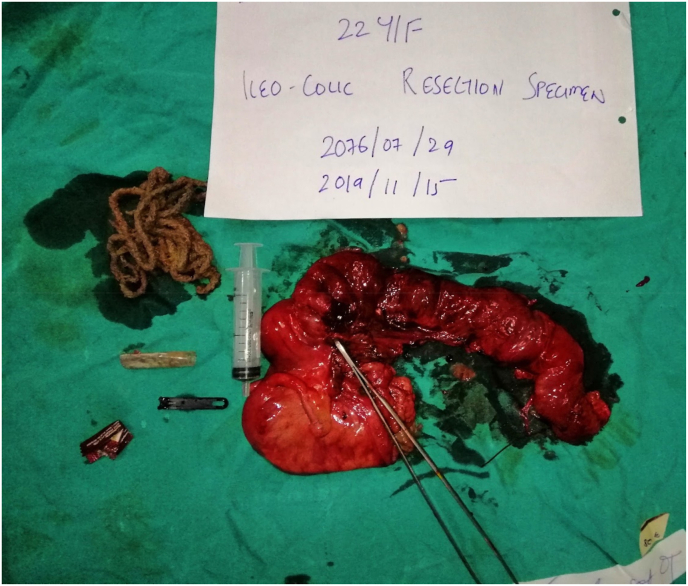
Resected postoperative ileocolic specimen with ingested foreign bodies including pieces of rope, a pen cap, and a candy wrapper.

She underwent emergency exploratory laparotomy which revealed dilated small bowel loops up to 40cm from the ileocecal valve. Adhesions were present in the region of the terminal ileum. An ileal perforation of approximately 2 × 1cm was found around 6cm proximal to the ileocecal valve where the intraluminal foreign body was discovered. Fig. 1. The bezoar included pieces of rope, a pen cap, and a candy wrapper lodged proximal to the ileocecal valve. Fig. 2. Limited ileocolic resection with end-to-back ileocolic anastomosis was performed. Fig. 2.

The patient also had a history of drug abuse with opiates. Therefore, psychiatric consultation was done but the evaluation revealed no psychiatric illness. The patient was discharged on fifth post-operative day with no complications. The patient was lost to follow-up.

## 3 Discussion

Bezoars are classified based on their composition as phytobezoars (non-digestible fibers of fruits and vegetables), trichobezoars (hair fibers), pharmacobezoars (medicines), lactobezoars (milk and curd) and miscellaneous bezoars or polybezoars (other less frequent materials) [6]. There are limited articles signifying the presence of foreign bodies leading to formation of bezoars. Moreover, bezoar found in a normal gastrointestinal tract is a rare condition.

Patients may be asymptomatic or present with findings suggestive of bowel obstruction, including abdominal distention, nausea, vomiting, or obstipation [7]. If presented late or if there is a delay in management, complications may arise including bowel perforation, gangrenous bowel, gastrointestinal bleeding, hypovolemia due to third space loss, and dyselectrolytemia due to vomiting [7].

Chaudhery et al. reported a similar case of small bowel obstruction and perforation secondary to a bezoar where emergency laparotomy was done and the affected bowel segment was resected [8]. In a case report by Shah et al., the patient had distended bowel loops due to phytobezoar with gangrenous changes that were resected followed with ileo-ileal anastomosis after a laparotomy [9]. Nasri et al. reported a case of foreign body bezoar where a pair of crumbled wrinkled almost desiccated light tan thin translucent plastic gloves were discovered [10]. A similar course of events followed for our patient, except that our patient had ingested pieces of rope, a pen cap, and a candy wrapper which led to the formation of a foreign body bezoar. Although the affected ileal segments were dilated and distended, gangrenous changes had not supervened.

Foreign body bezoars are commonly found in patients with psychiatric illness [9]. Alternatively, they are encountered in children, patients with pica, or mental retardation [11]. Although the history of substance abuse led us to anticipate a psychiatric illness in our patient, detailed evaluation yielded no psychiatric disorder.

## 4 Conclusion

Foreign body bezoar as a cause of small bowel obstruction is a rare clinical entity. Though unusual, clinicians must rule out a history of foreign body ingestion to avoid a delay in management as it can lead to pressure necrosis, gangrene, and perforation. For that matter, radiology could be helpful in making an early diagnosis. In addition, prompt surgical intervention must be done for a successful management.

## Ethical approval

Institutional ethical approval was not required to write this case report.

## Sources of funding

None.

## Author statement

SR: Concept, manuscript editing, performed the surgery, guarantor.

NS: Concept, performed the surgery, manuscript editing.

AK: Manuscript writing and editing.

MG: Manuscript writing and editing.

## Research registration

N/A.

## Guarantor

Dr Sujan Regmee.

Department of Surgery,

Kathmandu Medical College and Teaching Hospital,

Sinamangal, Kathmandu, Nepal.

## Consent

Written informed consent was obtained from the patient for publication of this case report and accompanying images. A copy of the written consent is available for review by the Editor-in-Chief of this journal on request.

## Provenance and peer review

Not commissioned; externally peer-reviewed.

## Patient perspective

The patient agreed to consent to the case report after explaining that her information would be de-identified, and her report would be published in a scientific journal to add to existing literature about the disease.

## Declaration of competing interest

No conflict of interest.
